# Physical, Mechanical, and Flammability Properties of Wood–Plastic Composites (WPC) Containing Beech-Wood Flour and Flame-Retardant Additives

**DOI:** 10.3390/polym16202944

**Published:** 2024-10-21

**Authors:** Yalçın Boztoprak

**Affiliations:** Technology Faculty Metallurgy and Materials Engineering, Marmara University, 34722 Istanbul, Turkey; yboztoprak@marmara.edu.tr

**Keywords:** beech wood, flame retardant, polypropylene (PP), biocomposite, masterbatch, wood–polymer composite (WPC)

## Abstract

This study aims to develop a recyclable, economical, and flame-retardant composite material using polypropylene, beech flour, tetrabromobisphenol A bis (TBBPA), and antimony trioxide (ATO). Flame-retardant additives (TBBPA and ATO) were initially added into polypropylene at different rates, and masterbatch (MB) samples were produced by the extrusion method. Subsequently, different percentages of wood flour (10%, 15%, 20%, 25%, and 30%) along with 60% MB were added to the polypropylene to create wood–polymer composites (WPC) using the injection method. The TBBPA, ATO, and wood flour were introduced through side-feeding hoppers during injection to ensure a homogeneous distribution within the WPC. Physical, thermal, and mechanical tests were conducted on the WPC samples. Additionally, TGA, FTIR, and SEM analyses were performed. The results indicated that the optimal ratios for TBBPA and ATO additives were 20% and 10%, respectively. It was observed that increasing the wood flour content in the WPC samples led to enhanced density, water absorption, hardness, impact, and abrasion resistance. Conversely, MFI, bending strength, and tensile strength decreased with higher wood flour content. It was observed that WPC samples exhibited flame resistance up to 725 °C. The produced WPC materials can be used in flooring applications, interior furniture, decorative wall panels, and aesthetic structural elements due to their fire behavior, good mechanical properties, low water-absorption rates, and aesthetic appearance.

## 1. Introduction

Natural fibers and wood flour used in biocomposite materials provide many advantages in terms of economy, sustainability, and environmental health. Biocomposites are materials derived from a biological source and contain one or more phases [1]. Reinforcing natural fibers, wood flour used as fillers, natural resins, and natural rubbers are widely used in biocomposite materials. These materials, found in nature, have high specific hardness and strength. They are also lightweight, cheap, renewable, recyclable, and biodegradable materials, so they are highly preferred in the composite industry [2]. In addition, these materials are alternative ecological materials that can be used in composite production due to their low density and satisfactory physical, mechanical, thermal, and aesthetic properties [3]. Additionally, they do not release a large amount of carbon dioxide into the atmosphere when burned. Natural fiber and particle-reinforced composites can be used in transportation (automobiles, train wagons, and aviation), military applications, the construction and building industries, packaging, and consumer products [4].

Composite materials made by incorporating wood as a natural fiber with a plastic matrix are called wood–plastic composites (WPCs). In studies related to thermoplastic matrix WPC materials, it is seen that various wood species, such as radiata pine, yellow pine, kenaf, red pine, western pine, spruce, and poplar, are used [5,6,7,8]. Wood particle decomposes at low temperatures. If the melting temperature of the matrix material exceeds the wood particle decomposition temperature, the wood particle will be thermally degraded by the elevated temperatures and lose its properties. Therefore, the matrix material should melt at a temperature below the wood particle decomposition temperature, preferably 200–220 °C. Since wood is an anisotropic material, it is susceptible to deformation, bending, and cracking due to the dimensional instability caused by changes in ambient humidity [9]. For this reason, the moisture absorption of the selected matrix material should not be high. Considering all these limitations, polypropylene, which has a suitable melting temperature, low water-absorption rate, and is resistant to living organisms such as bacteria and fungi, is generally preferred. Polypropylene is a promising matrix material for the formation of natural–synthetic polymer composites when combined with fibrous natural polymers suitable for admixtures and reinforcing elements [10].

In addition to extrusion and injection molding, studies are now being carried out on hot-pressing technology for plastic shaping of flat WPC sheets [11]. Yue et al. impregnated Chinese fir wood with boric phenol formaldehyde (BPF) resin at a concentration of 30% and then hot pressed it at different compression ratios. According to the test results, they stated that the higher the compression ratio, the better the mechanical properties and burning performance of the BPF-densified sample [12]. Cheng et al. modified poplar wood by combining impregnation of borate-containing phenol-formaldehyde resin and transverse compression of varying densities. They investigated the effects of this combined treatment on fire resistance. The test results showed that the combination of BPF resin impregnation and compression improves the fire resistance of poplar wood [13].

Polymer materials can readily burn when exposed to heat in an oxygenated environment. When WPC materials are exposed to direct sunlight, their covalent bonds can be damaged, in which case the mechanical properties are weakened, and they become brittle [14,15]. At the same time, bacteria and fungi can form rapidly within the wood fiber. This situation reduces the durability and lifespan of the wood. To prevent these issues and ensure the flame resistance of WPC materials, this study used tetrabromobisphenol A (TBBPA) as a flame-retardant additive due to its low toxicity, UV resistance, and antimicrobial activity. TBBPA is a white powder with a 67.7% Br content, suitable for processing temperatures of polypropylene and wood flour with a melting point of 113–178 °C [16]. Additionally, antimony trioxide (ATO) was used to increase the reaction rate of the TBBPA flame-retardant additive in this study. Antimony trioxide is a white powder additive with the chemical formula Sb_2_O_3_. It is commonly used as a synergist for brominated flame retardants, enhancing their effectiveness. Antimony trioxide is particularly effective in combination with aromatic products, improving flame resistance and helping to reduce the flammability of various materials. Its role in flame retardancy makes it valuable in applications such as plastics, textiles, and electronics, where fire safety is a critical concern [17,18].

Beech wood is known for its hardness, good mechanical properties, especially high compressive strength, strong fiber structure, and easy availability. Due to its durable structure, beech plywood is preferred in various applications, such as flooring, toy production, and furniture manufacturing. Therefore, beech wood (Fagus sylvatica), belonging to the Fagaceae family found in Turkey, was preferred in this study.

In their study, Çavuş and Mengeloğlu examined the melt flow index (MFI) and mechanical properties of the WPCs produced by adding wax, olive pomace, and red pine wood flour into PP. They found that there was no significant change in the melt flow index, but the density increased with the increase in the filler, and the filler reduced the tensile strength and elongation at break [19]. Arao et al. compared WPC materials with PP matrix, 50% wood flour, MAPP, and flame retardants such as ammonium polyphosphate (APP), melamine polyphosphate (MPP), and aluminum hydroxide in different proportions. They reported that PP containing 10% APP by weight increased self-extinguishing properties in the UL94 flammability test, while PP containing 30% APP by weight did not self-extinguish, and flame retardants caused a decrease in mechanical properties [20]. Pokhrel et al. produced WPC by using four different types of wood flour in a PP matrix and examined the mechanical properties of the resulting composites [21]. Bledzki and Faruk worked on the effect of wood filler geometry on the physical and mechanical properties of PP/wood flour WPC materials [22]. Stark and Berger investigated the effects of wood flour types and particle sizes on the mechanical properties of polypropylene containing wood flour. They reported that as the wood flour content increased, the tensile and bending modulus, density, heat-deflection temperature, and notched impact energy of the composites increased, while the tensile and bending strength, tensile elongation, mold shrinkage, melt flow index, and unnotched impact energy decreased [23]. Ito et al. stated that the mechanical properties of polypropylene-based WPC were improved by the addition of wood flour [24]. Ayrılmış et al. conducted a study in which they produced WPC by adding wood flour and olive cake into polypropylene. They used MA-g-PP (maleic anhydride-grafted polypropylene) to enhance compatibility between the matrix and the fillers. They noted that there was no significant change in the water resistance and bending properties of the WPC material, and they reported that the coupling agent had no effect on the bending strength [25]. Isa et al. worked on WPC materials containing wood flour. They found that the tensile and bending properties of composites containing wood flour were 10% higher than those of composite materials without wood flour [26].

In this study, the physical, mechanical, and flammability properties of wood–plastic composite (WPC) materials containing beech wood were investigated. Polypropylene (PP) was used as the matrix. Two flame retardants, TBBPA and ATO, were utilized, with ATO intended to enhance the effect of TBBPA. First, TBBPA and ATO were added to the PP in varying ratios to produce a masterbatch through an extrusion process. Then, wood flour and the masterbatch containing the optimal ratios of TBBPA and ATO were fed through different zones during the injection process to produce WPC samples. These procedures were carried out to ensure homogeneous distribution of the materials and to improve their flammability properties.

## 2. Materials and Methods

### 2.1. Materials

Homopolymer polypropylene matrix with high MFI was supplied from DUCOR Petrochemicals (Rotterdam, The Netherlands). Antimony trioxide (ATO) and tetrabromobisphenol A bis (TBBPA) were obtained from AMİK Italia (Milano, Italy). The particle size of ATO ranges between 100 and 900 nanometers, while the particle size of TBBPA ranges between 1 and 3 microns. Fine beech-wood flour (max. 180 μm in size), belonging to the Fagaceae family, was obtained from the Bayram Ticaret company located at the Keresteciler Industrial Site in Istanbul, Turkey. To determine the most suitable PP/TBBPA/ATO ratio, seven samples were preliminarily conducted and tested.

Since the density of the wood flour (WF) is low, even if it is mixed with the flame-retardant additives and fed into the extruder, the wood flour will remain on the walls under the effect of centrifugal force, and the flame-retardant additives will be fed into the machine first. Since a homogeneous structure would not occur, a masterbatch (MB) was produced. To prepare the masterbatch, samples were produced according to the ratios in Table 1. It was determined that the optimum TBBPA and ATO ratio in the MB should be PP/TBBPA/ATO 2 (20% TBBPA, 10% ATO), considering the results of the UL94 flammability and glow wire test.

Masterbatch (MB) was produced by adding 50% PP, 33.40% TBBPA, and 16.6% ATO. Thus, when 60% MB was added to the WPC, the TBBPA and ATO ratios in PP/TBBPA/ATO 2 were achieved. In addition, when the wood flour ratio was taken into account, it was concluded that the MB ratio in the WPC was appropriate.

WPC samples were produced by adding MB and wood flour into PP. While the masterbatch rate in WPC was determined as 60%, the remaining 40% consisted of PP and wood flour. The proportions of materials used to produce WPC samples are shown in Table 2.

### 2.2. Preparation of the Injection-Molded WPC Specimens

#### 2.2.1. Wood Flour-Drying Process

The strength of the obtained WPC samples can be negatively affected by the moisture content of the wood flour. In order to prevent this issue, the wood flour was subjected to drying. The appropriate drying temperature and time were calculated by a moisture test (see Section 2.4.1). The wood flour was dried at 140 °C for 15 min. The dried wood flour was cooled in a vacuum oven.

#### 2.2.2. Extrusion Process

**Figure 1:**
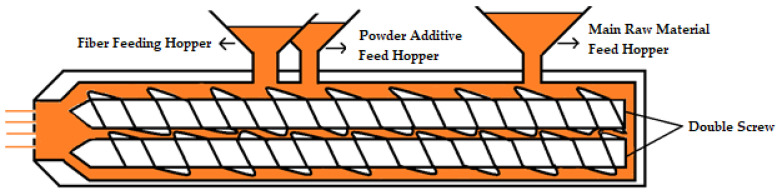
Double-screw extruder feed zones.

Granules were produced in a Coperion ZSK26 MC18 extruder machine. In the extruder, raw materials and additives were fed from different feeding hopper. TBBPA and ATO were mixed according to the specified ratios and then added from the powder-additive feed hopper of the extruder. Homopolymer polypropylene material was added from the raw material feed hopper of the extruder. Wood flour was added from the fiber-feeding hopper (Figure 1).

Twin screws were preferred for homogeneous mixing of compounds with different contents. The yield for each sample was determined to be 45 kg/h. The lowest possible temperature values were preferred to avoid burning or degradation of the wood flour. Since wood flour was fed from the heat 3 zone, the first two temperatures did not affect the sample properties. After the third temperature value of the extruder, the temperature of all WPC samples was kept constant. While the melt flow decreased as the amount of wood increased, the screw speed was increased in WPC 3, 4, and 5 to avoid production shortages and achieve homogeneous product output. The extruder parameters are shown in Table 3.

#### 2.2.3. The Injection Process

The PP/TBBPA/ATO granules were stored in an oven at 50 °C for 3 h before injection molding. WPC granules were dried in an oven at 100 ± 5 °C for 6 h. The dried granules were processed on the ARBURG 370 Allrounder injection-molding machine. The injection-starting temperature was 40 °C, back pressure 80 bar, screw rotation speed 35 rpm, and injection speed 80 cm^3^/s for all samples. The injection parameters are shown in Table 4.

### 2.3. Sieve Analysis

Sieve analysis was conducted using a vibrating sieve (Retsch, AS200, Haan, Germany) set to an amplitude value of 70 to determine the wood particle size/quantity ratio. Approximately 100 g of wood flour was placed on top and sieved through 1 mm, 180 µm, 90 µm, 63 µm, 53 µm, 45 µm, and 38 µm sieves for 15 min. The amount of dust remaining from the sieve analysis was calculated using Equation (1).

(1) Amount of dust remaining on the sieve=Mt−Mb

*Mt*: total weight of the powder and sieve.
*Mb*: empty sieve weight.

### 2.4. Physical Tests

#### 2.4.1. Moisture Test

A moisture test was performed to determine the moisture content in the wood flour and samples. Moisture content was calculated according to Equation (2).

(2) Moisture (%)=(My−Mk)/Mk×100

*My*: wet weight of the samples.
*Mk*: dry weight.

By keeping the wood flour in the oven at different temperatures and times, its moisture content was calculated according to Equation (2), and the optimum temperature and time were determined for fast production. Then, the WPC samples were dried in the oven at this determined temperature and time, and the moisture content was calculated according to Equation (2).

#### 2.4.2. Density Test

The density was measured with the Sartorius Analytic A120S (Göttingen, Germany) density tester to determine the density of the PP/TBBPA/ATO and WPC samples. In accordance with TS EN ISO 1183-1 Standard [27], three measurements were taken for each sample, and the average value was calculated.

#### 2.4.3. Water-Absorption Test

To determine the water-absorption rate of the WPC samples when used in contact with water, a water-absorption test was performed. Three samples of 1 mm and 2 mm thickness were subjected to the water-absorption test in accordance with ISO 62 Standard [28]. The samples were kept in distilled water at room temperature for 24 h and 48 h. Water-absorption rates were calculated according to Equation (3).

(3) Water−Absorption Rate (%)=(St−It)/It×100

*St*: final weight value.
*It*: initial weight value.

### 2.5. Melt Flow Index (MFI) Testing

A Zwick Roell-Mflow (Ulm, Germany) melt flow tester was used to determine the melt flow index of the granules. A high-flowability homopolymer polypropylene was used, as the wood particle would reduce the melt flowability. MFI values were obtained by applying a load of 2.16 kg at 190 °C in accordance with TS EN ISO 1133-1 Standard [29], cutting the melt once every 5 s. Three samples from each group were tested, and their averages were calculated.

### 2.6. Mechanical Properties

#### 2.6.1. Tensile Tests

Tensile tests of the samples were performed with an Instron 3367L4848 (Norwood, MA, USA, ABD) model tensile tester at constant room temperature. Tensile tests were conducted at a test speed of 50 mm/min for PP/TBBPA/ATO samples and at a test speed of 5 mm/min for WPC samples. Sample dimensions were determined as “type B” according to TS EN ISO 527-2 and TS EN ISO 3167 Standards [30,31]. Five samples were tested for each sample group, and their averages were taken.

#### 2.6.2. Impact Tests

The Izod notched impact test of the samples was conducted on the Zwick Roell HIT5.5P (Ulm, Germany) device. Ten samples produced with 80 × 10 × 4 mm dimensions in accordance with ISO 180 Standard [32] were tested. Izod notched samples were notched in “type A” geometry and dimensions. A 1 kJ pendulum was used. The impact strength was calculated by taking the average of the samples.

#### 2.6.3. 3-Point Bending Test

The 3-point bending test was carried out to determine the bending strength of the materials (Zwick, Z010, Ulm, Germany). In accordance with the ISO 178 Standard [33], impact test specimens with a thickness of 4 mm were placed so that the distance between the two supports was 64 mm. Five samples from each group were tested with a bending speed of 2 mm/min, and the values obtained were averaged.

#### 2.6.4. Hardness Tests

A hardness test was performed according to the Shore A method. The mean hardness of the sample was determined by calculating the arithmetic mean of the measurements at five separate measurements from the sample surface at least 6 mm intervals.

#### 2.6.5. Wear Test

The abrasion test of the materials was carried out with a Gotech GT-7012-T (Taichung, Taiwan) device. The abrasion tests of the samples were carried out with a Pin-on-Disk Testing Device with CS10 wheels, according to ASTM G99 Standard [34] under 250 gr weight load at 72 rpm speed. Weight measurements were taken every 50 m (50 m = 200 turns) for a total of 250 m (1000 turns). The weight loss in the material as a result of abrasion was calculated according to Equation (4), and the wear rate was calculated according to Equation (5).

(4) $$∆m=m_{1}-m_{2}$$

Δ*m* = weight loss (*g*)

(5) Ws=∆m/(ρ×Fn×L)

*Ws*: wear rate (cm^3^/Nm).
ρ: material density (gr/cm^3^).
*L*: wear distance (*m*).
*Fn*: applied load value (*N*).
*m*_1_: initial weight value.
*m*_2_: final weight value.

### 2.7. Flammability Tests

#### 2.7.1. UL94 Flammability Test

The UL94 test was carried out in the Info Protech UL94 (Midrand, South Africa) test chamber to determine the flammability properties of the samples. The UL94 test was carried out at 23 °C and 50% relative humidity after 48 h after molding. Three test samples with 0.8 mm, 1.6 mm, and 3.2 mm thickness were placed vertically according to the UL94 Standard [35].

#### 2.7.2. Glow Wire Test

The glow wire test (GWIT) was conducted in the cabin to determine the burning times and flame resistance of PP/TBBPA/ATO and WPC samples (Cabin; EMS, GW-2008, Ankara, Turkey). The GWIT test was conducted to determine the maximum temperature at which the samples did not ignite for more than 5 s. Samples with a thickness of 2 mm having dimensions in accordance with TS EN IEC 60695-2-10 and TS EN IEC 60695-2-13 Standards [36,37] were tested at 960 °C, 850 °C, and 750 °C.

### 2.8. Fourier-Transform Infrared Spectroscopy Analysis (FTIR)

Characterization of polypropylene, PP/TBBPA/ATO, and WPC samples was carried out using an Alpha Bruker FTIR spectrometer with ATR technology (Billerica, MA, USA). The samples were characterized by transmitting infrared rays in the range of 400–4000 cm^−1^ at room temperature.

### 2.9. Heat-Deflection Temperature (HDT) Test

The heat-deflection temperature (HDT) was determined by applying increasing temperature under constant load to the samples placed in the oil pool. The HDT test was performed to determine the effect of the wood flour contained in WPC samples according to the ISO 75-1 Standard [38] on composite material distortion temperature (HDT/Vicat S, Zwick Roell, Ulm, Germany). Two samples of impact bars of each WPC group were tested in the HDT device up to a temperature of 300 °C by exposure to a total load of 306 g in an oil bath with an initial temperature of 30 °C. Samples began to distort at 0.34 mm. The temperature value of 0.34 mm was calculated as the HDT temperature.

### 2.10. Thermogravimetric Analysis (TGA)

The TGA test was performed with a LiKrom TGAQ50 (Saugus, MA, USA) tester in accordance with the ISO 11358-1 Standard [39] to determine the decomposition temperatures of the materials, observe the effect of additives and wood flour on the decomposition temperature, and determine the final residue amount in the crucible. Weighed samples were heated at 0 °C to 1000 °C at a rate of 10 °C/min with a nitrogen gas flow of 50 mL/min.

### 2.11. Scanning Electron Microscopy (SEM)

Scanning electron microscopy (FEG SEM; FEI, Sirion XL-30, Tokyo, Japan) was used to examine the morphological characterization of the samples and to determine whether a homogeneous structure was formed. To make the samples conductive, the sample surfaces were coated with 10 Å thick gold/palladium alloy. PP/TBBPA/ATO 2, WPC 1, and WPC 5 samples were subjected to SEM analysis. Dimension analysis of wood flour in the WPC sample was performed with EDX system, and the change in size was observed.

## 3. Results and Discussion

**Figure 2:**
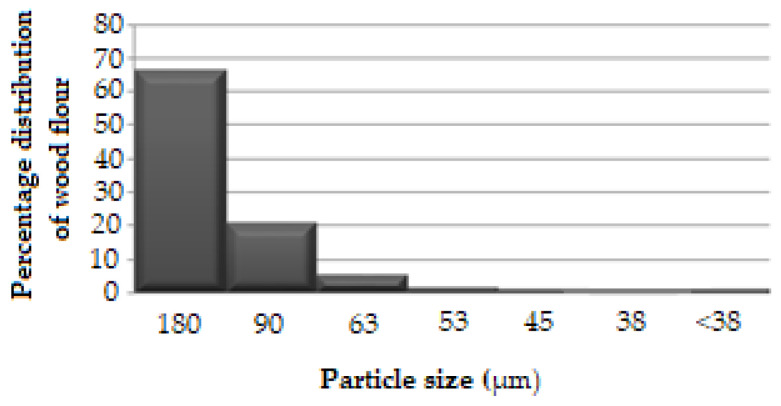
Wood flour distribution graphs.

Sieve analysis was performed to determine the particle size distribution of wood flour. The literature review showed wood flour particle sizes in the range of 50 to 700 μm were used in WPC production [40]. The beech-wood flour used in our study consists of particles with a maximum size of 180 µm. The graphs created according to the determined particle size/wood flour ratio value data are shown in Figure 2. The largest proportion was found in wood flour with the largest particle size (180 µm). As the particle size decreases, the wood flour ratio also decreases.

**Figure 3:**
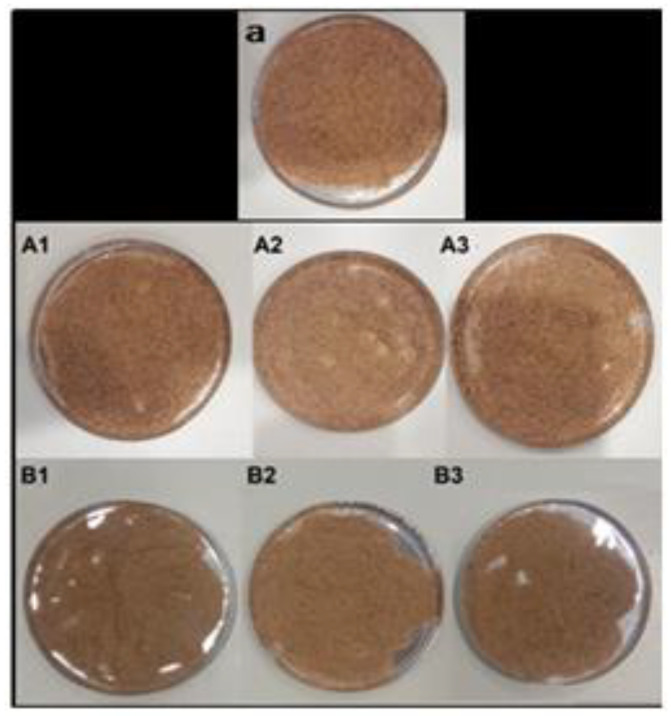
(**a**) Appearance of sample before drying; (**A1**–**A3**) 140 °C and (**B1**–**B3**) 120 °C; 1: 15 min, 2: 30 min, and 3: 60 min.

When wood flour was kept in the oven at 23 °C for 7 days, its moisture content was calculated to be 5.56%. In order to dry quickly, wood flour was kept in the oven at temperatures of 120 °C and 140 °C for 15, 30, and 60 min (Figure 3), and then, the optimum temperature and time were determined by calculating the moisture contents according to Equation (2). The standard deviation was not calculated because only one sample was used for each test. At this determined temperature and time, WPC granules were also subjected to a drying process, and their moisture content was calculated. Considering the applied temperature and time, 140 °C for 15 min was preferred for quick drying.

It was observed that the increase in temperature and time during the drying process was inversely proportional to the moisture content of the material, and the moisture content decreased depending on the increase in temperature and time (Table 5). It was also determined that the increase in temperature did not affect the color and appearance of the wood flour. The moisture content of the wood flour decreased significantly after the drying process. The moisture content of the WPC granules was calculated as 0.29%.

It was stated in the sources that the moisture content of unprocessed and oven-dried wood flour should be a maximum of 12% [41]. Based on this ratio, the moisture content values of wood flour and WPC samples are considered suitable.

The additives and fillers used in plastic materials are important components that affect the physical properties of the material. In this study, while the density of PP/TBBPA/ATO 2 was 1.1214 gr/cm^3^, the density of the WPC samples increased with the addition of wood flour (Table 6). The density of the WPC 1 sample with 10% wood flour added is 1.1767 gr/cm^3^, while the density of the WPC 5 sample with 30% wood flour added is 1.2737 gr/cm^3^. As can be understood from this, as the amount of wood flour increases, the density of the WPC samples also increases.

In the study by Çavuş and Mengeloğlu, where they used olive pomace and red pine wood flour in a PP matrix, it was observed that the density increased as the amount of wood flour increased [19]. The same result has been obtained and reported in many studies [42].

In WPC materials, water absorption may increase due to the wood flour or other filler materials they contain. Generally, water absorption increases as the exposure of the wood pores in the material to water and the exposure time increase.

The water-absorption rate of WPC samples was determined by the water-absorption test and calculated according to Equation (3). The standard deviation was not calculated because only one sample was used for each WPC group.

The sample thickness and the duration of water exposure are two important test parameters. It was observed that the amount of water absorption decreased with increasing sample thickness. Additionally, the samples exposed to water for 48 h absorbed more water than those exposed for 24 h.

As the amount of wood flour increased in WPC samples, the amount of water absorption increased (Table 7). The water-absorption rate of WPC 1, which was 1 mm thick and kept in water for 24 h, was 0.24%, while in WPC 5, this rate increased to 1.37%. This rate was 0.66% in WPC 1, which was kept in water for 48 h, and it increased to 2.18% in WPC 5. In samples with a thickness of 2 mm, as the amount of wood flour increased, the amount of water absorption also increased similarly. However, as the thickness increased, the water absorption showed a decrease. The percentage increase in water absorbed in B samples was less than that in A samples.

The melt flow index (MFI) indicates the workability of the material and its performance in the production process, making it one of the most important parameters in injection molding. It was seen that the MFI value of the matrix material, i.e., homopolymer polypropylene, increased with the addition of TBBPA and ATO additives.

The effect of these two additives on viscosity was clearly observed in PP/TBBPA/ATO 2. Thus, it was revealed that TBBPA [43] and ATO caused a decrease in viscosity, in other words, an increase in flowability. The addition of wood flour and the increase in the amount of wood flour in WPC samples decreased the flowability (Table 8). The MFI value of the PP/TBBPA/ATO 2 sample was 80.58 g/10 min, while it was 43.15 g/10 min for WPC 1 and 13.19 g/10 min for WPC 5.

In PP/TBBPA/ATO 2, which does not contain wood flour but contains only TBBPA and ATO, the tensile strength and % elongation ratio are higher; these values are 32.8 MPa and 8.61%, respectively. The elasticity modulus is the lowest; this value is 1681 MPa. It was determined that with the increase in the wood flour ratio in WPC samples, the tensile strength and the % elongation decreased, the elasticity modulus increased, and the samples showed brittle fracture. In the WPC 1 sample, where the wood flour rate is 10%, the tensile strength is 24 MPa, the modulus of elasticity is 1846 MPa, and the % elongation rate is 4.22, while in the WPC 5 sample, where the wood flour rate is 30%, the tensile strength is 20.4 MPa, the modulus of elasticity is 2602 MPa, and the % elongation rate is 2.33 (Table 9). Lignocellulosic wood fillers usually improve the tensile modulus of thermoplastic composites [44].

In the study conducted by Kurt and Mengeloğlu [45], it was reported that the tensile strength of the PP/pine wood flour/MAPP mixture with 25% ammonium polyphosphate (APP) as flame retardant was 15.68 MPa. In contrast, when compared to the WPC 5 sample without any compatibilizer, our sample showed a tensile strength of 20.4 MPa, indicating an approximately 33% higher value. This result revealed that the WPC 5 sample obtained without a compatibilizer is more advantageous in terms of tensile strength.

In the Izod impact test, the impact strength of PP/TBBPA/ATO 2 was found to be 1.82 kJ/m^2^, and it was determined that the impact strength increased depending on the addition of wood flour and the increase in the amount of wood flour. In the WPC 1 sample, the impact strength was 1.83 kJ/m^2^, while in the WPC 5 sample, this value increased to 1.98 kJ/m^2^ (Table 10). Wood has the capacity to absorb energy during impact. Thus, wood flour showed this effect.

In their study, Çavuş and Mengeloğlu found that the Izod (notched) impact strength of the composite material they produced by adding 20% red pine flour, wax, and MAPP to PP was 1.80 kJ/m^2^ [19]. In the WPC 3 sample, which contains the same amount of wood flour, the impact strength is higher at 1.86 kJ/m^2^.

Pokhrel et al. produced WPC materials using 80% PP and 20% of four different types of flour. They used white cedar, white pine, spruce fir, and red maple as wood flour. They found that the Izod impact strength of the WPC materials ranged from 0.018 to 0.023 kJ/m^2^ [21]. It is evident that higher impact strength results were obtained in this study, where beech-wood flour was used.

Since PP/TBBPA/ATO 2 has a more ductile structure compared to the WPC samples, it is an expected result that the amount of deflection is high, and the modulus of elasticity is low. The bending strength of the PP/TBBPA/ATO 2 sample was approximately 30% lower than that of the WPC samples. While the bending strength of PP/TBBPA/ATO 2 was 29.3 MPa, this value increased to 45.3 MPa in the WPC 1 sample. These results indicate that the addition of wood flour enhances the bending strength of the material. However, as the wood content increases, the samples become more rigid, which can lead to a decrease in bending strength. This increase in rigidity may also result in brittle behavior in the material. Consequently, while the bending strength of WPC 1 was determined to be 45.3 MPa, this value decreased to 41.3 MPa in WPC 5. Additionally, the increase in the amount of wood flour raised the modulus of elasticity and reduced deflection. In WPC 1, the elastic modulus was 1340 MPa, and the deflection was 5.2%, whereas in WPC 5, these values were 2140 MPa and 2.4%, respectively (Table 11). The lowest modulus value was observed in PP/TBBPA/ATO 2. This is due to the fact that lignocellulosic woody structures possess a higher elastic modulus than polymers [46,47].

In their study, Pokhrel et al. found that the bending strength of WPC materials was in the range of 40–45 MPa. Similar results were obtained in this study, where beech-wood flour was used [21].

When examining the PP/TBBPA/ATO 2 sample, it is evident that TBBPA and ATO have a significant effect on hardness. The hardness of the PP/TBBPA/ATO 2 sample, which does not contain wood flour, is higher than that of the WPC samples. This indicates that flame-retardant additives increase hardness more effectively than wood flour [2]. However, in general, the inclusion of wood components in polymer materials tends to increase hardness. Therefore, increasing the amount of wood flour in WPC samples led to an increase in hardness. The Shore A hardness of the PP/TBBPA/ATO 2 sample was 84.2, while the hardness values for the WPC 1 and WPC 5 samples were 75.8 and 81, respectively (Table 12).

The results obtained from the abrasion test are shown in Table 13. The weight loss and wear rate of the PP/TBBPA/ATO 2 sample are higher than those of WPC samples. The weight loss and wear rate of the PP/TBBPA/ATO 2 sample are 0.0017 g and 2.473 × 10^−6^ cm^3^/Nm, respectively, while these values are 0.0012 g and 1.755 × 10^−6^ cm^3^/Nm for the WPC 1 sample. Wood flour increased the abrasion resistance of WPC materials. As the amount of wood flour increased, the weight loss and wear rate decreased. In the WPC 5 sample, the weight loss and wear rate decreased and were 0.0007 g and 0.981 × 10^−6^ cm^3^/Nm, respectively. Materials with higher hardness generally tend to have better abrasion resistance, which is clearly seen here.

Ibrahim et al. produced WPC materials using the injection-molding method with wood flour (WF) at weight ratios of 5%, 15%, 25%, 35%, 45%, and 55%, incorporating malleated polypropylene. They achieved the lowest weight loss with a 60% reduction in WPC materials containing 25% wood flour compared to PP. In other words, while obtaining optimal wear resistance in WPC materials with 25% wood flour, they found lower values with higher wood flour content [48].

The UL94 test was conducted on all PP/TBBPA/ATO samples to determine the optimum ratio of flame retardants and on all produced samples to assess their flammability properties. UL94 test results are shown in Table 14. The UL94 test conducted on PP/TBBPA/ATO samples indicated that samples with thicknesses of 0.8 mm, 1.6 mm, and 3.2 mm did not burn, but all exhibited dripping, thus classifying them as V2. It was observed that they did not burn the cotton placed underneath. Additionally, it was found that as the sample thickness increased, the burning time and amount of dripping decreased.

Arao et al. produced WPC materials containing a PP matrix, 50% wood flour, MAPP, and ammonium polyphosphate (APP). They set the APP content at 10% and 30%. They reported that the material with 10% APP self-extinguished in the UL94 flammability test, while the material with 30% APP did not show self-extinguishing properties [20]. In the WPC samples in this study, it was observed that while the burning time increased, the amount of dripping decreased. Although samples WPC 1, 2, and 3 had burning times that qualified them for V0 classification, they were categorized as V2 due to dripping. For samples WPC 4 and 5, it was noted that, despite the flame being drawn, they continued to burn, and the cotton burned. Additionally, as the amount of wood flour in the WPC samples increased, the burning rate also increased.

The glow wire test was conducted on all PP/TBBPA/ATO samples to determine the optimum ratio of flame retardants and on all produced samples to assess burning time and flame resistance. The glow wire test results are shown in Table 15.

All PP/TBBPA/ATO samples and WPC 1, WPC 2, and WPC 3 samples extinguished as soon as the flame was withdrawn at 960 °C. It was observed that the samples dripped but did not burn the paper. It was determined that the WPC samples containing wood flour burned longer. It was determined that the WPC 4 and WPC 5 samples continued to burn for more than 30 s when the flame was withdrawn and burned the paper by dripping. WPC 4 and WPC 5 samples could not pass the 960 °C test temperature and 850 °C test temperature depending on the burning time. When the same samples were subjected to a temperature of 750 °C, it was observed that the samples did not burn but produced a significant amount of smoke. It was determined that as the total additive ratio of TBBPA and ATO increased, the burning temperature increased while the burning time decreased.

The GWIT test results indicate that samples 1 and 5 from the PP/TBBPA/ATO group extinguished flames at 725 °C, while samples 2, 3, and 4 extinguished at 875 °C. Samples 6 and 7, on the other hand, extinguished at 675 °C. This variation in flame-extinguishing temperatures suggests differing thermal stability and effectiveness of flame retardants among the samples. Such differences could be attributed to the specific compositions of each sample. It was observed that as the wood flour ratio increased, the amount of dripping decreased.

Both the UL94 and glow wire tests indicated that PP/TBBPA/ATO 2 provided the optimum results in terms of burning time and ignition temperature (GWIT) among the PP/TBBPA/ATO samples. Therefore, the flame-retardant additive ratios in the production of WPC samples were based on the PP/TBBPA/ATO 2.

Candan et al. conducted a study on the fire performance of laminated veneer lumber (LVL) panels treated with different fire-retardant chemicals. They treated beech veneers using a mixture of borax and boric acid as well as monoammonium phosphate and diammonium phosphate. The fire test results revealed that the LVL panels treated with diammonium phosphate exhibited the lowest ignition temperature at 220 °C, while the panels treated with a borax–boric acid mixture reached the highest ignition temperature at 420 °C [49]. On the other hand, the WPC samples containing TBBPA and ATO obtained in this study showed a flame resistance of up to 725 °C, offering a significantly higher flame resistance than LVL panels.

**Figure 4:**
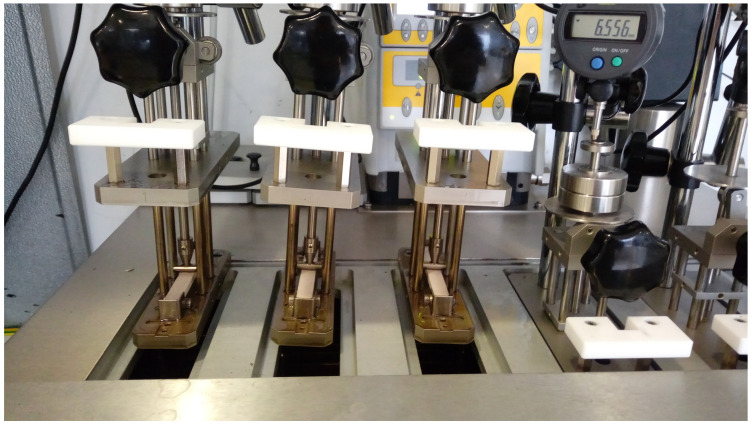
The placement of samples in the HDT device.

Determining the heat-deflection temperatures (HDT) of the samples plays a critical role in determining the usability areas of plastic and composite materials. WPC materials containing a high percentage of wood flour delay degradation and provide better thermal stability to the composite material. This makes the material more durable and long-lasting in various applications. For this reason, the HDT-A test was applied to all WPC samples (Figure 4). It was determined that the heat-deflection temperatures of the samples increased with the increase in the wood flour content of the WPC samples (Table 16). While the heat-deflection temperature of the WPC 1 sample containing 10% wood flour was 72.28 °C, this temperature increased to 86.71 °C in the WPC 5 sample containing 30% wood flour. Increasing hardness generally enhances the material’s resistance to heat, which can lead to a rise in the heat-deflection temperature. As can be seen from the results, such an increase was observed here.

**Figure 5:**
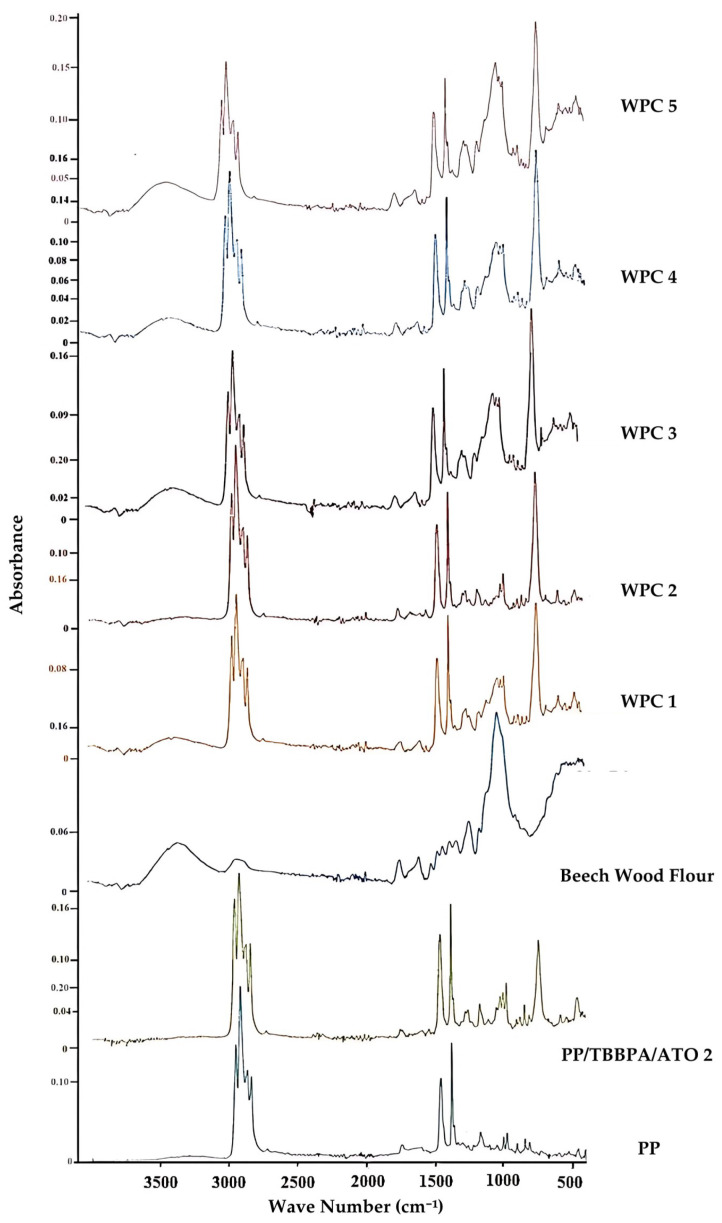
FTIR graphs of PP, wood flour, PP/TBBPA/ATO, and WPC samples.

FTIR analysis revealed the characteristic region distributions of the materials (Figure 5). In the FTIR examination of homopolymer polypropylene, peaks were observed at 2950 cm^−1^ and 2910 cm^−1^ for the methyl group (CH_3_); at 2870 cm^−1^, 2840 cm^−1^, and 1460 cm^−1^ for the methylene group (CH_2_); and at 1375 cm^−1^ for CH_3_, while a CH_2_ peak was noted in the range of 400–1000 cm^−1^ [50].

In the FTIR analysis of TBBPA, peaks were identified at 1551 cm^−1^ (C=C−X), 1475 cm^−1^, 1393 cm^−1^ (symmetric bending of CH_3_), 1318 cm^−1^ (out-of-plane deformation of OH), 1278 cm^−1^ and 1243 cm^−1^ (in-plane deformation of OH), 1159 cm^−1^ (C−OH), and 737 cm^−1^ (aromatic group CH stretching), and the vibrations in the range of 500–700 cm^−1^ correspond to C−Br stretching [51]. When comparing the PP/TBBPA/ATO samples with PP, an increase in peaks in the 1000–1400 cm^−1^ range was observed. This increase can be attributed to the chemical presence of TBBPA and ATO additives as well as the contribution of the metal hydroxide content.

The FTIR analysis of wood flour showed a peak for hydroxyl (OH) in the 3000–3500 cm^−1^ range, a characteristic peak for CH_4_ in the 2800–3000 cm^−1^ range, carbonyl functional group double peaks in the 1650–1750 cm^−1^ range, and vibrations related to C−O−C and C−C stretching frequencies between 1000–1500 cm^−1^ [52]. When examining the FTIR spectrum of the WPC sample, it was observed that, although not as sharp as that of wood flour, there was still a hydroxyl (OH) peak in the 3000–3500 cm^−1^ range. Additionally, the peaks in the 1650–1750 cm^−1^ range exhibited a broader trend due to chemical interactions, resulting in reduced sharpness. Conversely, the peaks in the 500–1500 cm^−1^ range showed increased sharpness and formed composite peaks as a result of these chemical interactions.

**Figure 6:**
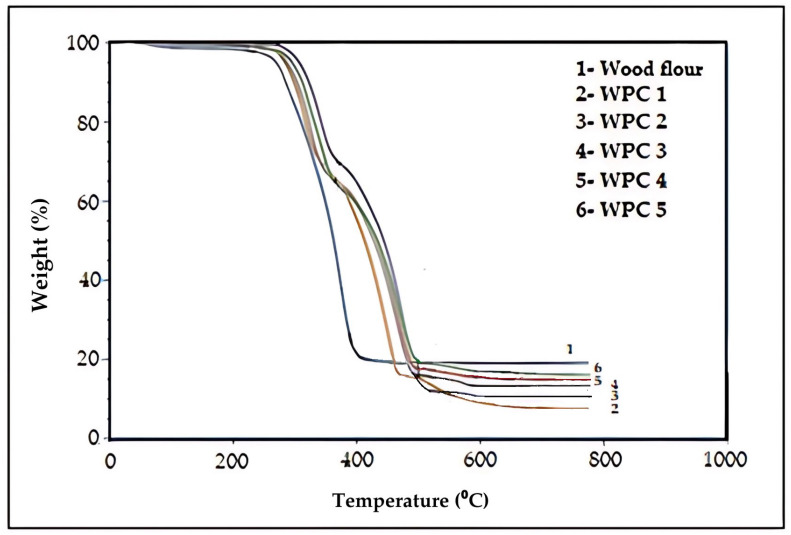
TGA graphics of wood flour and WPC samples.

According to the TGA analysis results in Figure 6, it was determined that wood flour began to decompose at 278 °C and reached its maximum degradation temperature at 400 °C. Lignocellulosic materials such as wood and plants are composed of cellulose, hemicellulose, and lignin [53]. Studies have reported that hemicelluloses decompose in the temperature range of 275–350 °C, lignin at 360 °C, and cellulose in the range of 350–400 °C [54,55].

For polypropylene (PP), the initial degradation temperature was found to be 283 °C, while the maximum degradation temperature was 455 °C [56,57]. When examining the TGA graph of the WPC samples, it was observed that the addition of flame-retardant additives increased the initial degradation temperature of wood flour, as degradation occurred at 301 °C. Additives with aromatic rings, such as TBBPA, tend to decompose at higher temperatures due to their strong bonding. While pure wood decomposes rapidly, in WPC samples, the presence of additives results in a delay in degradation, corresponding to a weight loss in the range of 60% to 70%. Furthermore, it was observed that as the amount of wood flour increased, the final residue amount also increased. The residue amount was 6% in WPC 1 and 17% in WPC 5 (Table 17).

**Figure 7:**
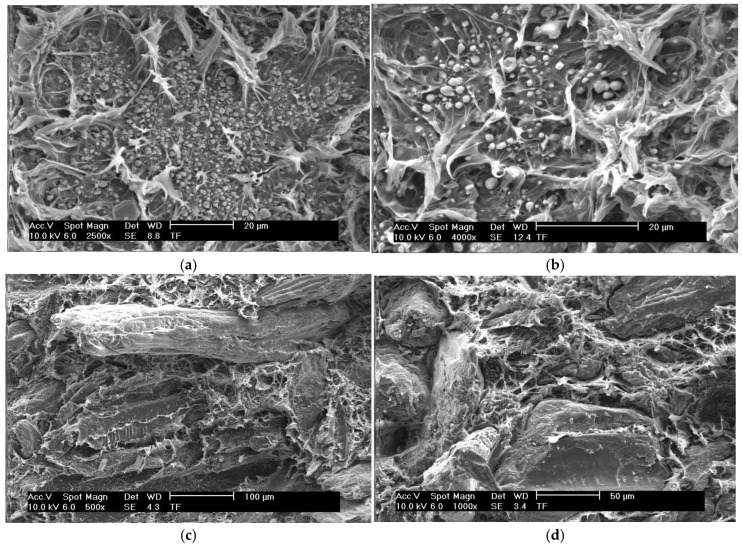
SEM images. (**a**,**b**) PP/TBBPA/ATO 2 and (**c**,**d**) WPC 5.

The SEM images of the PP/TBBPA/ATO 2 (a and b) and the WPC 5 (c and d) samples are shown in Figure 7. The particle sizes of ATO range from 100 to 900 nm, while TBBPA’s particle sizes are between 1 and 3 microns. In the SEM image shown in Figure 7b, small particles are observed that belong to ATO, whereas the larger particles are associated with TBBPA. The SEM images in Figure 7c,d show the beech-wood flour within the PP matrix.

In the SEM images of the PP/TBBPA/ATO 2 sample (a and b), a homogeneous distribution of flame-retardant additives is observed. This is an important factor that enhances the flame-resistance performance of the WPC material. In the SEM images of the WPC 5 sample (c and d), a homogeneous distribution is achieved, and a strong interfacial interaction between PP and wood flour is noted. This strong interaction plays a crucial role in improving the mechanical properties and durability of the material. The production process significantly affected these characteristics.

## 4. Conclusions

In this study, the physical, mechanical, and flammability properties of wood–plastic composite (WPC) materials containing beech-wood flour and flame-retardant additives (TBBPA and ATO) were investigated. Based on the current study, the following important conclusions were drawn:

1. All WPC samples were categorized under the V2 classification in the UL94 flammability test. In particular, WPC 1, WPC 2, and WPC 3 qualified for V0 classification based on their burning times but were classified as V2 due to dripping. All WPCs demonstrated flame resistance up to 725 °C according to the glow wire test, which is a favorable result for a WPC product. The addition of TBBPA and ATO effectively increased the flame resistance of the WPC;
2. The addition of wood flour reduced tensile strength, bending strength, and elongation while increasing density, water absorption, hardness, impact strength, and wear resistance;
3. As the amount of wood flour increased, an increase in heat-deflection temperature (HDT) was also observed. This indicates that the addition of wood flour enhances the thermal stability of the WPC material;
4. A higher percentage of wood flour reduced the flexibility of the materials, which caused them to exhibit more brittle behavior;
5. The SEM images showed that the flame-retardant additives and wood flour were homogeneously distributed. This result indicates that the practices involved in masterbatch production and the manufacturing process significantly influenced the uniform distribution, optimal mechanical properties, and enhancement of flame resistance;
6. The aesthetic appearances of WPC materials, along with their mechanical, flammability, and physical properties, make them ideal for use in interior furnishings, flooring, and decorative structures.

## Figures and Tables

**Table 1:** Material ratios used to prepare pre-production and masterbatch (MB) samples.

Sample	PP (%)	TBBPA (%)	ATO (%)
PP/TBBPA/ATO 1	77.5	15	7.5
PP/TBBPA/ATO 2	70	20	10
PP/TBBPA/ATO 3	62.5	25	12.5
PP/TBBPA/ATO 4	70	22.5	7.5
PP/TBBPA/ATO 5	70	15	15
PP/TBBPA/ATO 6	85	10	5
PP/TBBPA/ATO 7	92.5	5	2.5
MB	50	33.4	16.6
MB in WPC (60%)	30	20	10

**Table 2:** Material ratios used to prepare WPC samples.

			MB (60%)		
Sample	PP	WF	PP (%)	TBBPA (%)	ATO (%)
WPC 1	30	10	30	20	10
WPC 2	25	15	30	20	10
WPC 3	20	20	30	20	10
WPC 4	15	25	30	20	10
WPC 5	10	30	30	20	10

**Table 3:** The extruder parameters.

Heat (°C)	PP/TBBPA/ATO	MB	WPC 1	WPC 2	WPC 3	WPC 4	WPC 5
Heat 1	180	190	190	200	200	200	200
Heat 2	180	200	200	220	220	220	220
Heat 3	170	200	190	190	190	190	190
Heat 4	170	200	180	180	180	180	180
Heat 5	170	170	170	170	170	170	180
Heat 6	170	170	170	170	170	170	180
Heat 7	160	170	170	170	170	170	180
Heat 8	160	170	170	170	170	170	170
Heat 9	160	170	170	170	170	170	170
Heat 10	160	180	180	180	180	180	180
Mold Temperature (°C)	160	160	160	160	160	160	160
Melting Temperature (°C)	146	154	150	152	154	154	156
Screw Speed (rpm)	400	400	500	500	520	530	550

**Table 4:** The injection parameters.

Sample	Heat 1	Heat 2	Heat 3	Heat 4	Injection Nozzle Temp. (°C)
PP/TBBPA/ATO	170	170	170	170	170
WPC Samples	175	180	185	190	195

**Table 5:** Time–humidity values of wood flour at 120 °C–140 °C.

**120 °C**	**Initial Weight (g)**	**Final Weight (g)**	**Moisture (%)**
15 min	4.658	4.368	6.63
30 min	4.761	4.475	6.39
60 min	4.684	4.404	6.35
**140 °C**	**Initial Weight (g)**	**Final Weight (g)**	**Moisture (%)**
15 min	4.648	4.372	6.31
30 min	4.747	4.480	5.95
60 min	4.666	4.462	4.57

**Table 6:** Density of the specimens.

Sample	Density (g/cm^3^)
PP/TBBPA/ATO 2	1.1214 ± 0.004
WPC 1	1.1767 ± 0.002
WPC 2	1.2131 ± 0.005
WPC 3	1.2335 ± 0.007
WPC 4	1.2558 ± 0.006
WPC 5	1.2737 ± 0.002

**Table 7:** A: Water-absorption percentages of WPC samples with a thickness of 1 mm. B: Water-absorption percentages of WPC samples with a thickness of 2 mm.

A	Initial Weight (g)	Weight after 24 h (g)	Water-Absorption Amount (%)	Weight after 48 h (g)	Water-Absorption Amount (%)
WPC 1	1.65	1.65	0.24	1.66	0.66
WPC 2	1.78	1.79	0.33	1.80	0.84
WPC 3	1.86	1.87	0.48	1.88	1.07
WPC 4	1.87	1.90	1.27	1.91	1.81
WPC 5	1.97	1.99	1.37	2.01	2.18
**B**	**Initial Weight (g)**	**Weight after 24 h (g)**	**Water-Absorption Amount (%)**	**Weight after 48 h (g)**	**Water-Absorption Amount (%)**
WPC 1	3.55	3.55	0.11	3.55	0.19
WPC 2	3.78	3.79	0.13	3.80	0.42
WPC 3	3.90	3.92	0.46	3.94	1.12
WPC 4	3.64	3.66	0.57	3.68	1.18
WPC 5	3.84	3.86	0.59	3.91	1.69

**Table 8:** MFI value table of samples.

Sample	MFI (g/10 min)
PP	32.96 ± 0.92
PP/TBBPA/ATO 2	80.58 ± 0.18
WPC 1	43.15 ± 0.20
WPC 2	39.64 ± 0.40
WPC 3	31.91 ± 0.15
WPC 4	24.91 ± 0.10
WPC 5	13.19 ± 0.20

**Table 9:** Table of elasticity modulus, tensile strength, and elongation values of samples.

Sample	Elasticity Modulus (MPa)	Tensile Strength (MPa)	Elongation (%)
PP/TBBPA/ATO 2	1681 ± 26.19	32.8 ± 0.46	8.61 ± 0.63
WPC 1	1846 ± 13.20	24.0 ± 0.14	4.22 ± 0.32
WPC 2	2027 ± 25.91	22.6 ± 0.21	3.59 ± 0.18
WPC 3	2249 ± 27.60	21.7 ± 0.26	3.40 ± 0.18
WPC 4	2489 ± 54.60	21.4 ± 0.27	2.95 ± 0.23
WPC 5	2602 ± 61.00	20.4 ± 0.33	2.33 ± 0.17

**Table 10:** Impact strength of samples.

Sample	Izod Impact (kJ/m^2^)
PP/TBBPA/ATO 2	1.82 ± 0.23
WPC 1	1.83 ± 0.22
WPC 2	1.86 ± 0.08
WPC 3	1.86 ± 0.04
WPC 4	1.90 ± 0.15
WPC 5	1.98 ± 0.11

**Table 11:** Elasticity modulus, bending strength, and deflection values of samples.

Sample	Elasticity Modulus (MPa)	Bending Strength (MPa)	Deflection (%)
PP/TBBPA/ATO 2	562 ± 78	29.3 ± 1.2	6.9 ± 0.2
WPC 1	1340 ± 40	45.3 ± 0.5	5.2 ± 0.7
WPC 2	1516 ± 75	45.1 ± 1.3	4.0 ± 0.4
WPC 3	1728 ± 108	43.9 ± 1.0	3.2 ± 0.3
WPC 4	1936 ± 94	42.4 ± 0.6	2.8 ± 0.2
WPC 5	2140 ± 160	41.3 ± 0.9	2.4 ± 0.3

**Table 12:** Hardness values of samples.

Sample	Shore A Hardness
PP/TBBPA/ATO 2	84.2 ± 0.8
WPC 1	75.8 ± 3.8
WPC 2	79.8 ± 1.8
WPC 3	80.6 ± 1.6
WPC 4	80.8 ± 1.8
WPC 5	81.0 ± 1.0

**Table 13:** Weight loss and wear rates of PP/TBBPA/ATO 2 and WPC samples.

Sample	Weight Loss (g)	Wear Rate (cm^3^/Nm)
PP/TBBPA/ATO 2	0.0017 ± 1	2.473 × 10^−6^ ± 0.14
WPC 1	0.0012 ± 3	1.755 × 10^−6^ ± 0.30
WPC 2	0.0012 ± 2	1.524 × 10^−6^ ± 0.22
WPC 3	0.0011 ± 2	1.498 × 10^−6^ ± 0.22
WPC 4	0.0009 ± 1	1.169 × 10^−6^ ± 0.13
WPC 5	0.0007 ± 3	0.981 × 10^−6^ ± 0.30

**Table 14:** UL94 values of samples.

Sample	0.8 mm	1.6 mm	3.2 mm	Dripping
PP/TBBPA/ATO 1	V2	V2	V2	✓
PP/TBBPA/ATO 2	V2	V2	V2	✓
PP/TBBPA/ATO 3	V2	V2	V2	✓
PP/TBBPA/ATO 4	V2	V2	V2	✓
PP/TBBPA/ATO 5	V2	V2	V2	✓
PP/TBBPA/ATO 6	V2	V2	V2	✓
PP/TBBPA/ATO 7	V2	V2	V2	✓
WPC 1	V2	V2	V2	✓
WPC 2	V2	V2	V2	✓
WPC 3	V2	V2	V2	✓
WPC 4	V2	V2	V2	✓
WPC 5	V2	V2	V2	✓

**Table 15:** GWIT and GWFI temperature values and dripping properties of samples.

	PP/TBBPA/ ATO 1	PP/TBBPA/ ATO 2	PP/TBBPA/ ATO 3	PP/TBBPA/ ATO 4	PP/TBBPA/ ATO 5	PP/TBBPA/ ATO 6	PP/TBBPA/ ATO 7	WPC 1	WPC 2	WPC 3	WPC 4	WPC 5
GWFI (°C)	960	960	960	960	960	960	960	960	960	960	750	750
GWIT (°C)	725	875	875	875	725	675	675	725	725	725	725	725
Total Burning Time (s)	15	9	6	8	13	20	24	30	30	32	60	100
Dripping	√	√	√	√	√	√	√	√	√	√	√	√

**Table 16:** The heat-deflection temperature values of the WPC samples.

Sample	HDT- A (°C)
WPC 1	72.28 ± 0.80
WPC 2	75.57 ± 0.15
WPC 3	82.25 ± 1.15
WPC 4	83.54 ± 3.50
WPC 5	86.71 ± 1.80

**Table 17:** TGA analysis results.

Sample	Initial Decomposition Temperature (°C)	Maximum Decomposition Temperature (°C)	Residue at 800 °C (%)
PP	283	455	6
Wood Flour	278	400	16
WPC 1	308	462	6
WPC 2	302	494	8
WPC 3	301	487	12
WPC 4	301	490	14
WPC 5	304	489	17

## Data Availability

The original contributions presented in the study are included in the article.
